# Investigating Flow-Induced Corrosion of Magnesium in Ophthalmological Milieu

**DOI:** 10.3390/ma17174404

**Published:** 2024-09-06

**Authors:** Marco Ferroni, Francesco De Gaetano, Dario Gastaldi, Matteo Giuseppe Cereda, Federica Boschetti

**Affiliations:** 1 Chemistry Materials and Chemical Engineering Department “Giulio Natta”, Politecnico di Milano, 20133 Milan, Italy; marco.ferroni@polimi.it (M.F.); francesco.degaetano@polimi.it (F.D.G.); dario.gastaldi@polimi.it (D.G.); 2 MgShell S.r.l., 20133 Milan, Italy; 3 Eye Clinic, Fatebenefratelli and Sacco Hospital, 20157 Milan, Italy; matteo.cereda@gmail.com

**Keywords:** magnesium, ocular device, flow-induced shear stress, corrosion

## Abstract

Although the impact of local fluid dynamics in the biodegradation of magnesium is well known, currently no studies in the literature address the degradation effects of ocular vitreous on bioresorbable devices made of magnesium, which could be developed as drug delivery carriers. The aim of this study was to investigate the flow-induced corrosion mechanism of magnesium in an ophthalmological environment for future applications in ophthalmic drug delivery. To achieve this, experimental and computational methods were combined. Specifically, a CFD model was employed to design experimental conditions that replicate the ocular flow-induced shear stress (FISS) on manufactured magnesium samples. Pure Mg samples were tested in a bioreactor system capable of imposing the ocular CFD calculated values of FISS on the Mg samples’ surface by varying the pump flow rate. Optimal flow rates for a range of different FISS values specific to the ophthalmological fluid dynamics affecting the device were indeed determined before running the experiments. After conducting customized corrosion tests, morphological observations and profilometric maps of the eroded surfaces of Mg samples were obtained using scanning electron microscopy (SEM) and confocal laser scanning microscopy (CLSM). These maps were then post-processed for the parametric evaluation of corrosion rates. Pre-existing localized superficial defects did affect the final corrosion pattern. SEM images and CLSM data confirmed a uniform corrosion mechanism, with corrosion rates of 1.9, 2.7, and 3.4 μm/day under different shear stress conditions (0, 0.01, and 0.032 Pa, respectively). More generally, uniform corrosion on pure Mg samples increased with higher FISS values, and at higher shear stress values (FISS = 0.032 Pa), a notable washing-out effect of the corrosion products was observed. The removal of corrosion products at higher shear stresses suggests that the dynamic ocular environment, influenced by saccadic movements, plays a significant role in the corrosion mechanism of pure magnesium. The corrosion rates determined in this study, in conjunction with clinical drug release requirements, are crucial for designing potential drug-release devices for ocular applications.

## 1. Introduction

Magnesium (Mg) is a critical element that plays a pivotal role in regulating numerous physiological processes and preserving the integrity of various biological tissues [1,2,3]. Its integration into medical devices is highly advantageous, given its natural absorbability in biological fluids and the biocompatibility of its corrosion products [4,5,6,7,8,9,10,11,12]. These properties allow magnesium to address the limitations of polymeric matrices and other biomaterials. The pioneering use of magnesium in cardiovascular stents demonstrated its superiority as a biomaterial in the biomedical field [13,14,15,16,17]. Moreover, magnesium can be a viable option for temporary implants applications, particularly for orthopedic ones [5,18,19]. Extensive research underscores the impact of local fluid dynamics in the biodegradation of magnesium, including the specific types of corrosion, degradation rates, and resultant corrosion products [20,21,22,23,24,25,26,27,28]. The literature also highlights the need to analyze the effect of alloying elements on the corrosion behavior of pure Mg related to the content of impurities and magnesium processing history [29,30]. Magnesium is highly concentrated in ocular regions, such as the vitreous and aqueous humors, where it is essential for maintaining the structural and functional integrity of ocular tissues. Its role is particularly crucial in retinal function. Cataract and various diseases of the conjunctiva, choroid, and retina are directly linked to low values of magnesium [31,32,33,34].

Glaucoma is a leading ocular disease that could be effectively treated with an intraocular bioresorbable magnesium-based device [1]. Additionally, age-related macular degeneration (AMD), a chronic and degenerative condition affecting the macula, represents another strong candidate for magnesium-based intervention. AMD is the primary cause of vision loss in developed countries among people over 50 years old, with wet-AMD being marked by the overproduction of vascular endothelial growth factors (VEGF) that induce choroidal neovascularization [35,36,37,38]. While intravitreal injections of anti-VEGF drugs can stabilize or improve vision in patients, this treatment method is plagued by significant drawbacks, including poor patient adherence and a heightened risk of endophthalmitis [39,40,41,42,43]. Compliance with therapy is a major issue in the field of chronic and degenerative diseases, with 39.8% of patients worldwide abandoning their clinical regimen, resulting in underdosing due to fear and anxiety associated with injections for wet-AMD [44,45]. Patients need fewer injections, fewer appointments, and consistent visual outcomes. Therefore, the development of new sustained-release devices that can extend therapeutic intervals is essential to address these clinical challenges. Magnesium and its alloys are promising candidates for creating a new generation of intraocular bioresorbable drug delivery devices. However, to fully understand the behavior of these systems in the complex physiological environment of the eye, the unique corrosion mechanisms must be studied.

Currently, no studies in the literature address the degradation effects of ocular vitreous on bioresorbable devices made of magnesium. Fluid dynamics significantly influence the bioerosion process of pure Mg and Mg alloys, enhancing corrosion mechanisms, protecting material surfaces differently under various shear stress conditions, and preventing the accumulation of Mg products [20,21,22,23,24]. The gradual dissolution and temporary scaffolding of absorbable magnesium-based stents have garnered research and clinical interest due to the fluid drag forces acting on vessels [14,46,47,48]. Some researchers have investigated the corrosion behavior of magnesium and its alloys in dynamic conditions outside the human body, analyzing how magnesium alloys degrade in implanted physiological environments, and finding that degradation rates are much higher under flow conditions compared to static ones [27,28,49,50].

In our previous study (Ferroni et al. [51]), we provided a parametric description of the flow-induced shear stress (FISS) fields created by the liquefied vitreous in an injectable magnesium-based device. Therefore, the focus of this study is to describe the corrosion behavior of magnesium, considered a promising material in biomedical fields, particularly in ophthalmology [1]. Another critical aspect emphasized in the literature is the development of in vitro testing methods that can partially simulate in vivo conditions, identifying the local parameters that affect corrosion behavior while considering the human body as a complex system [23,25,49,50,52,53,54]. Moreover, while some studies have combined finite element simulation with experimental analysis to investigate flow-induced corrosion [8,28], none have focused on pure magnesium under dynamic ocular conditions.

Given this context, this study aims to investigate the corrosion mechanisms affecting magnesium samples, considering the unique rheological properties of the ocular milieu. The proposed method involves a combined numerical and experimental approach, which can be broken down into the following main steps:

- Manufacture of pure magnesium samples specifically designed for testing in an ocular bioreactor;
- Numerical determination of experimental flow rate conditions correlated to the flow-induced shear stress field;
- Development of an ocular bioreactor capable of applying appropriate fluid dynamic conditions;
- Evaluation of corrosion behavior characteristics using sample-specific morphology and profilometry.

The numerical method enables the accurate definition of experimental working conditions and the recreation of flow-induced shear stress fields described in the previous study [51]. This allows for the development of a tailored experimental setup capable of inducing various levels of shear stress on constrained magnesium samples, which is essential for studying the material corrosion mechanism in an ocular environment. Wang et al. [8] proposed a similar approach by inducing different shear stress levels on magnesium samples for corrosion tests, though their setup and imaging techniques differ from those in the proposed study. They used a rotating disk electrode system to induce fluid flow over the samples, evaluating corrosion behavior with electrochemical techniques like potentiodynamic polarization curves and electrochemical impedance spectroscopy. The corroded samples’ surface morphology was analyzed using scanning electron microscopy (SEM).

In contrast, the proposed study employs an ocular bioreactor to replicate the fluid dynamics of the vitreous humor and assesses the corrosion mechanism using morphological and profilometric images of free-eroded surfaces obtained through optical microscopy. Once the optimal inflow condition is achieved, specific corrosion tests on pure magnesium samples are conducted. Morphological and profilometric images and data of the free-eroded surfaces are acquired and processed to evaluate corrosion rates under both static and dynamic conditions. A comprehensive analysis of kinematic corrosion phenomena is performed, focusing on corrosion products, surface-profile modifications, and corrosion rates over time under different flow-induced shear stress configurations.

The objective of this study is to quantify the corrosion mechanisms affecting magnesium samples within the unique ocular environment. The main aim was to evaluate the impact of ocular fluid motion, particularly the shear stress generated by saccadic movements, on magnesium corrosion rate. By examining the corrosion behavior of magnesium under various shear stress conditions, the study aims to provide valuable insights for the development of effective magnesium-based drug-release devices. 

Indeed, in these devices, it is essential to evaluate the corrosion rate of the material as this directly influences the drug release kinetics.

## 2. Materials and Methods

### 2.1. Pure Magnesium Sample Preparation

To prepare the samples for the corrosion tests, pure magnesium bars were cut into 46 rectangular plates with dimensions of 9.78 ± 0.05 mm in length, 2.94 ± 0.07 mm in width, and 1.53 ± 0.04 mm in thickness from an extruded rod, following the same parameters as described in Liu et al. [55]. Specifically, magnesium bars were machined into small hollow billets, and then extruded at 350 °C with a specific extrusion rate. After cutting the extruded bar into parallelepipeds, the samples were then progressively ground using silicon carbide (SiC) paper up to 3000 grit and cleaned with acetone and alcohol via ultrasonic cleaning for 10 min to remove any contaminants.

### 2.2. Computational Model

A set of computational models was developed to numerically define: (i) the experimental conditions for corrosion testing, (ii) the design of the test chambers, and (iii) the evaluation of fluid dynamics within the chambers under experimental conditions.

The first computational analysis focuses on optimizing the chamber design by varying flow rate values. To achieve this, a parametric sweep analysis was employed to solve a sequence of steady-state problems, using a 1-parameter sweep (i.e., flow rate).

**Figure 1:**
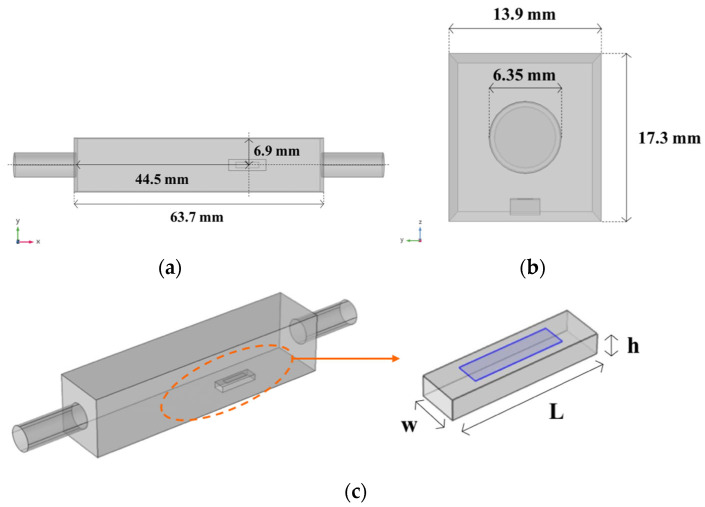
CAD sketches of the reference chamber for corrosion tests, where a pure Mg sample is placed: (**a**) top view showing the relative position of a constrained sample inside the chamber, and (**b**) front view along the direction of the flow. (**c**) Zoom of the pure magnesium sample constrained inside the chamber. The blue represents the target zone, where the averaged shear stress on the sample is computed, avoiding edge effects.

First, the domain geometry and placement of a single sample to be eroded were defined. The dimensions of the chamber and the constrained magnesium sample, in terms of length (L), width (w), and thickness (h), are summarized in Figure 1. The sample and chamber sizes were optimized to ensure a uniform distribution of flow-induced shear stress (FISS) in the target zone on the sample (blue area, zoom in Figure 1) while maintaining laminar flow within the volume (Re < 2000).

Hybrid meshes were created using 158,136 tetrahedral and prism elements, with a mesh quality of 0.71. Given the importance of computing an optimal FISS field on the target area of the tested sample, additional mesh refinement was performed, achieving a high average element quality of 0.94. The subdomain of the connection tubes was also refined using a swept mesh technique with an element quality of 0.82. PARDISO was used as the implicit stationary solver for the parametric sweep analysis. The medium inside the chamber has the same properties as the liquefied vitreous simulated in our previous studies [51,56,57], so the continuity and Navier–Stokes equations for a 3D steady-state incompressible Newtonian flow were implemented and solved using the commercial software COMSOL Multiphysics 5.3 (COMSOL Inc., Burlington, MA, USA). The imposed boundary conditions reflect the in vitro experiment, with inlet and outlet conditions set to ensure a numerically well-posed problem, describing a net flow into and out of the domain using the same two equations:

L_entr_∇_t_ ∙ [−p**I** + μ (∇_t_ **u** + (∇_t_ **u**)^T^)] = −p_entr_ **n**, (1)

L_exit_∇_t_ ∙ [−p**I** + μ (∇_t_ **u** + (∇_t_ **u**)^T^)] = −p_exit_ **n**, (2)

where L_entr_ [m] and L_exit_ [m] are the entrance and exit lengths of fictitious channels (>>0.06Re⋅D, where D is the hydraulic diameter) necessary for the full development of the laminar flow profile, p_entr_ [Pa] is the entrance pressure related to the imposed parametric flow rate Q [m^3^/s], and p_exit_ [Pa] is the exit absolute pressure. Additionally, a wall no-slip condition, where the fluid velocity relative to the walls is zero, was imposed on all other boundaries. Twelve different flow rate values (100, 150, 200, 250, 300, 350, 400, 500, 600, 700, 800, and 1000 mL/min) were applied in the parametric sweep by the device in the eye [51]. For this purpose, a SNOPT (Sparse Nonlinear OPTimizer) gradient-based method was chosen as the optimization algorithm. This method is suitable for this application as it serves as a general-purpose solver capable of handling large-scale problems more efficiently than derivative-free solvers. Gill et al. [58] provide a comprehensive description of the method. To function effectively, this method requires the initialization of appropriate objective and control variables. In this study, the objective and control variables were related to the target and flow rate (Q), respectively, with the aim of determining a unique flow rate (Q) that induces an averaged FISS on the sample target area (blue area, zoom in Figure 1). Additionally, the two variables were conveniently scaled to work within the range Ω ∈ [0,1] to facilitate the algorithm’s convergence towards the optimized solution. The objective variable was defined using the following expression, which needed to be minimized:

10^7^ ⋅ (τ_comp_ − τ_target_)^2^, (3)

where τ_comp_ [Pa] is the averaged FISS value computed on the target area for each iteration, whereas τ_target_ [Pa] was imposed as initial parameter. The multiplicative factor 10^7^ is related to the algorithm scaling. In order to scale the variable Q, a control parameter Q_control_ was introduced as follows:

Q = 1000^Qcontrol^, (4)

Equation (4) allows for the optimization of the flow rate value (Q) to be imposed for minimizing Equation (3), by working on a control parameter (Q_control_) defined in the range of Ω ∈ [0,1]. Reliable initial conditions for the variables involved in an optimization algorithm need to be defined to ensure solution convergence. For this reason, an initial value of the control variable Q_0_ was evaluated for each imposed FISS target using the computed Q-FISS characteristic curve. Starting from the relation between Q and Q_control_ (Equation (4)), an initial scaled control parameter Q_control0_ was defined as follows:

Q_control0_ = log_10_ Q_0_/3, (5)

All iterative simulations achieve convergence when an optimality tolerance of 10^−3^ is met, with a maximum of 10^3^ model evaluations. Using COMSOL Multiphysics, the previously proposed model was integrated with this optimization component. As a result, the geometry, meshing grid, governing equations, and boundary conditions remained consistent with those implemented earlier. Additionally, a parametric sweep of the main averaged FISS values, as detailed in our prior publication [51], was incorporated to fully automate the optimization process, from the initial fluid dynamic analysis to defining the experimental flow rate conditions for a given set of FISS values.

The model was thoroughly evaluated in terms of (i) numerical convergence within the feasible region, (ii) Reynolds number and velocity field during the operation of the setup, and (iii) the relationship between the applied flow rate (Q) and the flow-induced shear stress (FISS) on the exposed surface of the constrained sample.

### 2.3. Corrosion Experimental Setup

A specialized bioreactor was created to replicate ophthalmological stress conditions, specifically the flow-induced shear stress experienced by an injected drug delivery device. Pure magnesium samples were used to investigate the corrosion behavior, focusing on corrosion rate (CR), corrosion type, and corrosion products.

**Figure 2:**
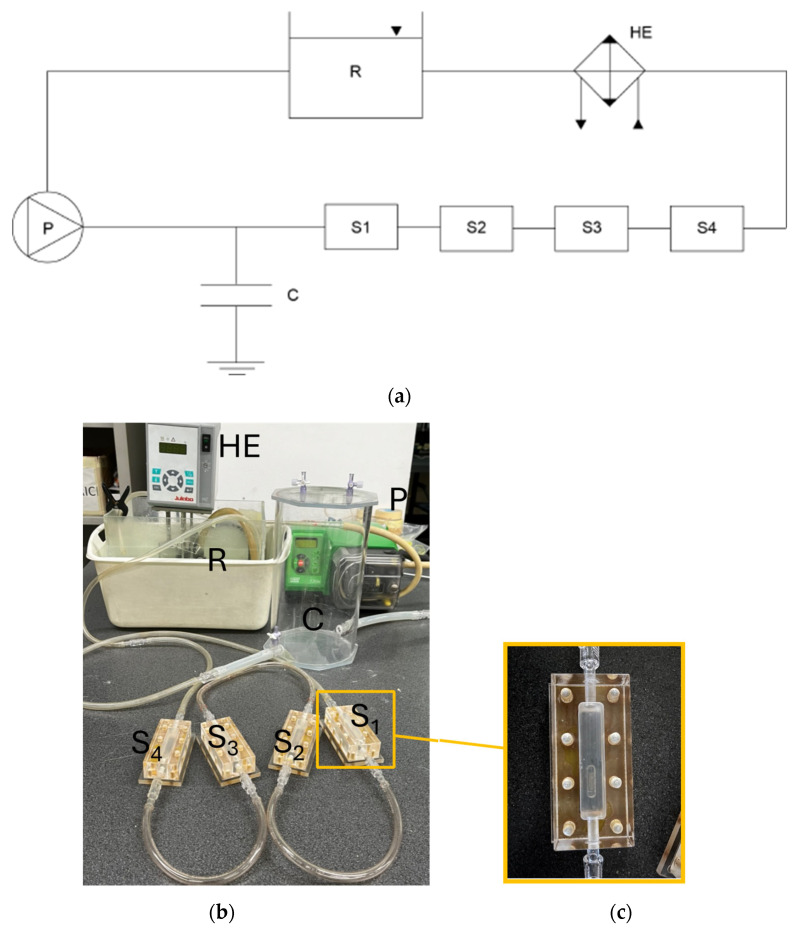
(**a**) Sketch of the experimental setup for corrosion tests, showing the peristaltic pump (P), the air compliance chamber (C) that minimizes flow pulsatility, the four chambers (S1, S2, S3, and S4) holding the Mg samples, the heat exchanger (HE), and the free surface reservoir (R). (**b**) Photograph of the experimental setup used for the corrosion tests. (**c**) Close-up view of one of the reference chambers where one of the Mg samples is placed.

The setup was partially based on the approach developed by Wang et al. [8] for their vascular bioreactor designed for Mg alloy and stent corrosion simulations. The ocular bioreactor for pure Mg samples included a mini-roller peristaltic pump, test sample chambers, corrosion medium, compliance components, a reservoir, and a heat exchanger (Figure 2). The peristaltic pump (520Du/R2, Watson-Marlow Ltd., Falmouth, Cornwall, UK) operated within a range of 0.1–220 rpm (0.70–1500 mL/min) using compliant tubing with a diameter of 6.35 mm. The flow rate set by the peristaltic pump at various rpm was verified using a transit-time ultrasound flowmeter (HT110 series, Transonic Systems, Ithaca, NY, USA). The test chambers were designed based on the numerical results from previous computational models and manufactured using Poly(methyl methacrylate) (PMMA, Plexiglas^®^).

Demineralized water, maintained at an ocular temperature of approximately 34 °C, was selected as the medium to simulate the highly liquefied vitreous rheological conditions associated with wet-AMD [59]. To minimize fluctuations caused by the roller pump, an air compliance chamber was installed upstream of the test chambers. This setup utilized 1/4” silicon tubing to connect the four chambers housing the samples. The heat exchanger, positioned within the free surface reservoir, maintained the corrosion medium at 34 °C.

Each sample was placed in a groove on the custom-designed PMMA supports, which were covered with a layer of cyanoacrylate-based glue, right after cleaning them with demineralized water and acetone for 5 min before being put in the four test chambers. Corrosion tests were conducted at intervals of 8, 24, and 48 h to analyze the corrosion mechanisms. The medium level was kept consistent throughout the tests.

Averaged flow-induced shear stress values of 0.032 and 0.01 Pa, representing two different saccadic conditions obtained from our previous study [51], were replicated on the sample surfaces by applying flow rates of 245 and 159 mL/min, respectively, based on numerical results. Static conditions (FISS = 0 Pa) were also tested as a control for comparison with dynamic stimulation.

### 2.4. SEM and CLSM Analysis

The morphologies compositions of pure magnesium samples, both before and after corrosion tests, were examined using a field emission scanning electron microscope (SEM, Zeiss EVO 50 EP, LaB6 cathode electron gun, Zeiss, Jena, Germany). The samples were analyzed at magnifications ranging from 75× to 2000× to assess overall topology and finer details. Prior to analysis, the magnesium samples were ultrasonically cleaned in demineralized water and acetone for 5 min.

Profilometric evaluation was conducted with a confocal laser scanning microscope (CLSM, Olympus LEXT OLS4100, Olympus, Tokyo, Japan) that provides high-resolution 3D imaging. A 5× magnification BF Plan Semi Apochromat objective lens (MPLFLN5X, Olympus, Tokyo, Japan) was used, offering a 2560-320 μm field of view, a 20.0 mm working distance, and a 0.15 numerical aperture. Additionally, a 10× magnification objective (MPLFLN10X, Olympus) was employed for detailed imaging, with a 1289-160 μm field of view, an 11.0 mm working distance, and a 0.30 numerical aperture. The in-plane (x;y) resolution was 120 nm, and the out-of-plane (z) resolution was 10 nm, facilitated by a 405 nm laser and high-aperture objective.

The 3D images were captured using a stitching technique, which analyzes a user-defined matrix of 3 × 7 cells from the bottom of the microscope plate to the top surface of the sample. The CLSM provided two types of information: (i) reflected intensity images, which contrast different materials’ surfaces, and (ii) height contrast images, which offer topographical reconstruction of the sample surface. Corrosion rates [μm/day] were calculated assuming uniform corrosion, though the presence of corrosion pits was also considered. Thus, corrosion rates depend on the reduction of magnesium volume on each tested sample and were calculated assuming uniform corrosion acting on the sample surface parallel to flow direction and exposed to controlled FISS as follows:

CR_i_ = ∆h_i_/∆t, (6)

where ∆h_i_ [μm] represents the decrease in height associated with the loss of magnesium volume, and ∆t [day] denotes the specific corrosion exposure time. The .csv files from all acquisitions were processed using custom scripts developed in a numerical environment (MATLAB R2018, MathWorks^®^, Natick, MA, USA).

This analysis aimed to provide a detailed characterization of corrosion by comparing the profiles, topologies, and morphologies of the samples before corrosion tests to assess their initial patterns, and then after 8, 24, and 48 h of exposure.

### 2.5. Statistical Analysis

For the study, a total of 40 pure Mg samples were used: 20 samples for FISS = 0.032 Pa, 16 samples for FISS = 0.01 Pa, and 4 samples for static control. The experimental results are expressed as means ± standard errors (SE), with all error bars in the figures representing the standard errors. Statistically significant differences were assessed using an unpaired *t*-test for normally distributed isolated pairs, and one-way analysis of variance (ANOVA) was employed to evaluate the uniform corrosion rates and the corresponding decrease in sample height with increasing FISS. Differences were deemed significant if *p* < 0.05. All the statistical analyses were performed using scripts developed in MATLAB.

## 3. Results

### 3.1. Computational Analysis of the Fluid Dynamic inside the Chamber

**Figure 3:**
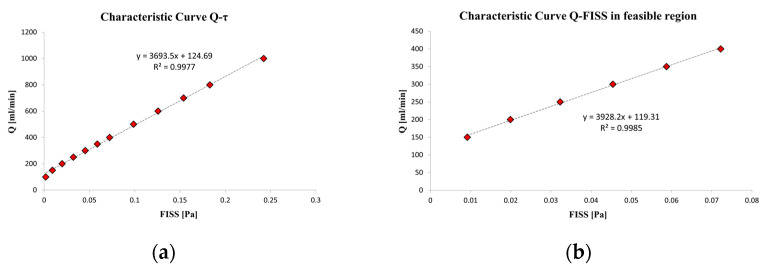
(**a**) Characteristic relation between the defined flow rate Q and the induced averaged shear stress FISS on the target area of the sample. The linear interpolation curve properly fits the parametric data from 100 to 1000 mL/min. (**b**) Zoom of the characteristic relation between flow rate Q and induced averaged shear stress FISS on the target area of the sample in the feasible region. The best fitting can be linearly represented as well.

Figure 3 (left panel) illustrates the relationship between flow rate (Q) and the average flow-induced shear stress (FISS) on the target area of the sample. The Q-FISS curve can be accurately interpolated using a linear relationship between these two variables (R² = 0.9977). The feasible region, defined by the minimum and maximum shear stress values simulated across different configurations detailed in Ferroni et al. [51], is highlighted in Figure 3 (right panel) and also interpolated with a linear fit (R² = 0.998).

**Figure 4:**
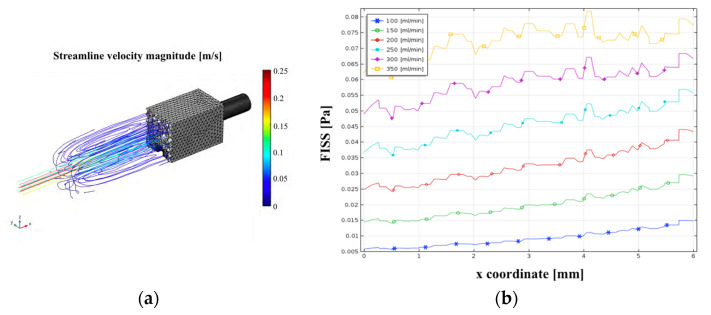
(**a**) Map of the streamline velocity magnitude for a steady-state analysis inside the chamber for a flow rate Q imposed equal to 250 mL/min. (**b**) Centerline plots of the flow-induced shear stress FISS along the flow direction (from left to right) on the target areas for different inlet flow rates in the feasible region (100, 150, 200, 250, 300, and 350 mL/min).

Additionally, a steady-state streamline analysis was conducted to visualize the velocity magnitude inside the chamber (Figure 4, left panel) and to plot the shear stress along the flow direction at the centerline (Figure 4, right panel). For clarity, only the streamline analysis for Q = 250 mL/min and the shear stress profile trends for flow rates within the feasible region were presented.

Lastly, Reynolds numbers were calculated for each parametric configuration using Equation (8) from Ferroni et al. [56] and are presented in Table 1. This was done to confirm the laminar flow regime for all imposed flow rates, ensuring the absence of turbulence that could affect the corrosion mechanisms and their uniformity. The Reynolds number evaluation was performed on the cut plane across the sample, where the reference velocity was the averaged velocity magnitude, and the characteristic length was the width of the chamber.

### 3.2. Numerical Definition of the Corrosion Experimental Conditions

**Figure 5:**
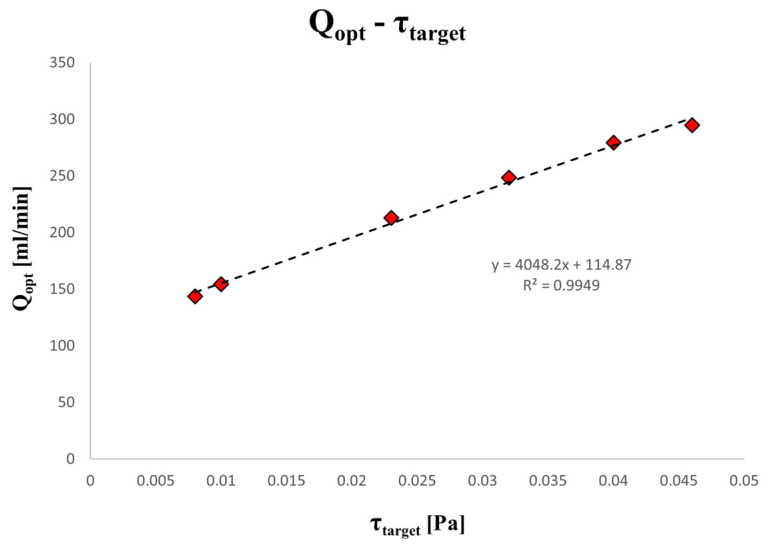
Characteristic relation between the flow rate Q to impose and the FISS field on the target area defined as target in the optimization process. The linear interpolation curve properly fits the parametric data from 0.008 to 0.046 Pa.

First, the performance results of the optimization method are presented in Table 2, which includes three random FISS values as targets (0.01, 0.03, and 0.05 Pa). The results demonstrated high accuracy and low computational costs, as evidenced by the minimal difference between the target and computed FISS values. Following this, Table 3 displays the corresponding optimal flow rates (Q_opt_) for a range of different FISS values specific to the ophthalmological fluid dynamics affecting the device. Their linear relationship is illustrated in Figure 5. Specifically, the six FISS values considered are related to the profiles discussed in Ferroni et al. [51], which characterize the varying impacts of ocular movements on intravitreal fluid dynamics.

### 3.3. Imaging Outcomes and Statistical Profiles of Corrosion Rate

**Figure 6:**
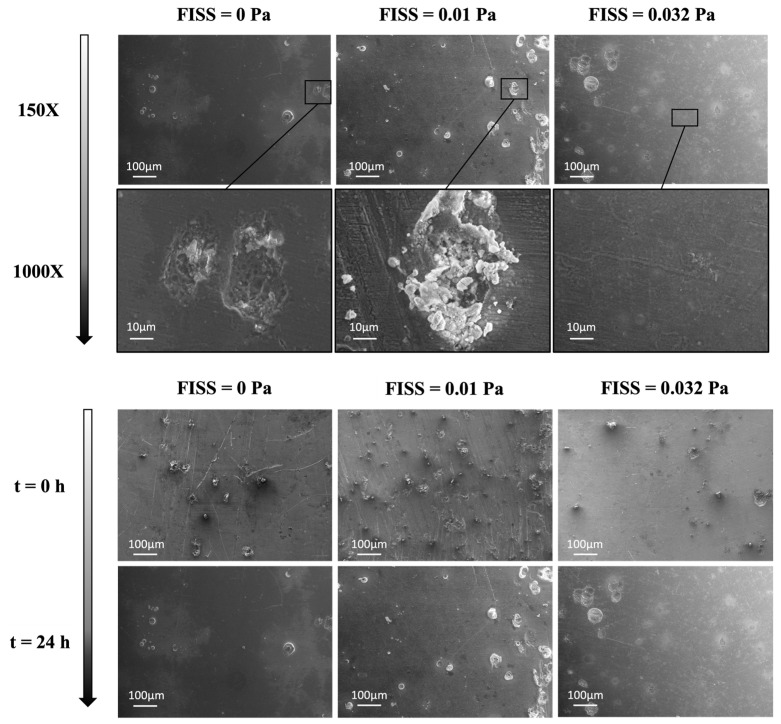
(Top panel) Three different morphologies that can be observed at FISS values of 0, 0.01, and 0.032 Pa, respectively after 24 h of test. From left to right, at different magnifications and scales: pitting corrosion resulting in the formation of holes, typical corrosion product magnesium oxide (MgO), and flat uniform corrosion. (Bottom panel) 24 h time-scale analysis of the sample morphology; localizations are already present before testing (t = 0). After 24 h of fluid flow, magnesium localizations are oxidized and the resulting corrosion products are eroded, resulting in a flat surface. All the pictures of the bottom panel are 150× magnification.

Figure 6 (top panel) illustrates the corrosion morphologies of pure Mg samples in the ocular bioreactor under FISS values of 0, 0.01, and 0.032 Pa over one day. The macro-morphologies indicate a general pattern of localized corrosion, with three distinct scenarios highlighted, occurring randomly in each FISS configuration. The first zoom (FISS = 0 Pa) reveals a crack, the second (FISS = 0.01 Pa) shows a pitting corrosion product, and the third (FISS = 0.032 Pa) highlights extensive flat regions with uniform corrosion. The presence of pitting corrosion products on multiple sample surfaces was examined by comparing macro-morphologies before and after each experimental test. Figure 6 (bottom panel) displays similar patterns across entire surfaces at different time intervals, indicating that the presence of localizations is not linked to the overall corrosion behavior. A few instances of external contamination were observed during the experiments and identified through SEM images.

**Figure 7:**
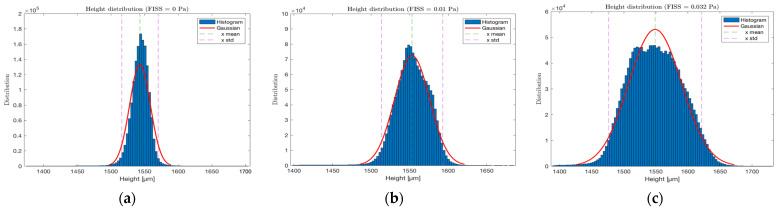
Gaussian distribution of typical local height after corrosion test. Mean and standard deviation values of the normal distribution are reported for FISS equal to (**a**) 0 Pa, (**b**) 0.01 Pa, and (**c**) 0.032 Pa.

The 3D macroscopic corrosion profiles and topologies were generated from CLSM data of pure Mg samples exposed to FISS values of 0, 0.01, and 0.032 Pa for corrosion periods of 8, 24, and 48 h in the ocular bioreactor. The CLSM data were post-processed using custom scripts to distinguish between the plate, support, and sample levels, enabling the assessment of local height profiles for each sample after a corrosion time step. Figure 7 presents histograms showing the normalized distribution of local heights for samples subjected to different FISS fields (0, 0.01, and 0.032 Pa). The histograms reveal a normal distribution in terms of the local height reduction, indicating uniform corrosion when FISS fields are applied to pure Mg samples.

**Figure 8:**
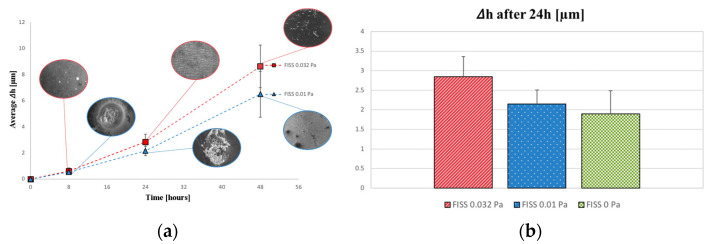
(**a**) The trend in the average height decreases over time after 8, 24, and 48 h of corrosion tests is shown. During the first 8 h, there is no significant difference between (i) FISS = 0.01 Pa and (ii) FISS = 0.032 Pa (one-way analysis of variance (ANOVA) test, *p* < 0.05). (**b**) The amount of height variation in the Mg samples after 24 h of corrosion is shown. A one-way ANOVA test (*p* < 0.05) indicated no significant difference between static and dynamic conditions with FISS equal to 0.01 Pa.

Height profiles and corresponding corrosion rates for various FISS values and time intervals were analyzed. Figure 8 (left panel) presents the average height decrease profiles over time for FISS values of 0.032 and 0.01 Pa. A detailed view of the 24 h simulation data, comparing height decreases with those observed under static conditions (FISS = 0 Pa), is shown in Figure 8 (right panel).

**Figure 9:**
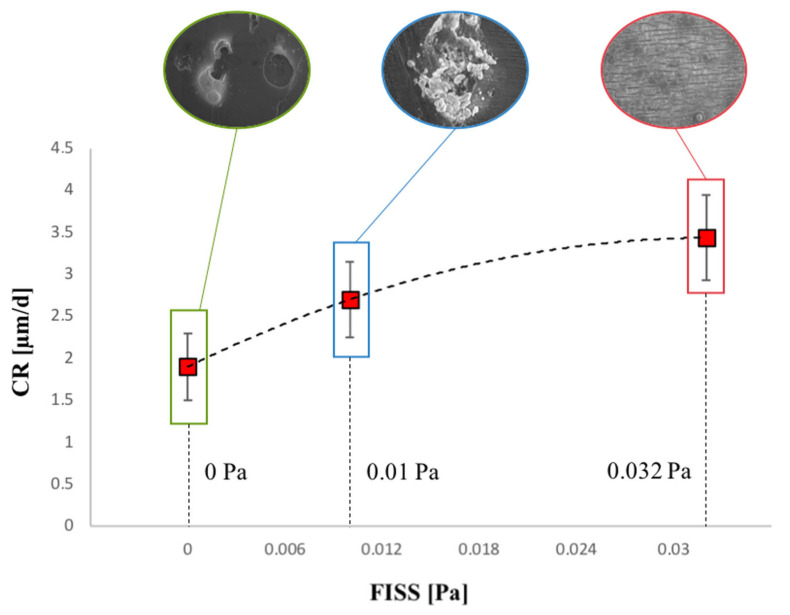
Trend and bar graph of corrosion rate (CR) of Mg degradation varying the FISS values. The first derivative of the CR trend tends to decrease to zero as the analysis moves from a static to the most stressful condition analyzed (0.032 Pa).

Corrosion rates (CR) were assessed using data from 24 and/or 48 h of testing, as these were deemed the most reliable. Figure 9 illustrates the CR trends across the three tested FISS fields. Volume loss (VL) percentages were calculated for each FISS configuration considering the reduction in thickness, maintaining length and width constant, and extended over a 7-day period. The results indicate volume losses of 1.7%, 1.13%, and 0.88% for FISS values of 0.032 Pa, 0.01 Pa, and 0 Pa, respectively, with the formation of an Mg oxide layer on the exposed surfaces considered in the VL evaluation.

## 4. Discussion

The influence of fluid dynamics on magnesium corrosion has been examined, with a focus on how fluid movement and rheology impact corrosion reactions [60]. This study specifically explores how fluid flow shear stress in the ocular system affects corrosion mechanisms in pure Mg samples. It highlights the kinetics and types of corrosion through numerical modeling, morphological analysis, and profilometric evaluation.

A key outcome of the fluid dynamic analysis inside the chamber is the convergence of the solution across the entire feasible region defined by the parametric sweep, avoiding simulation singularities. Additional insights from the parametric sweep include detailed fluid dynamic conditions within the chambers where magnesium samples are held. The relationship between flow rate (Q) and FISS, shear stress along flow direction centerlines, and Reynolds number across all parametric configurations were analyzed. A linear relationship between Q and FISS was established, providing a valuable tool for setting initial values for the control and objective variables in the optimization method. Establishing reliable initial conditions for these variables is crucial for solution convergence and managing computational costs.

Wang et al. [8] employed a similar approach to assess flow-induced shear stress, though their sample chamber differed significantly. They observed FISS values along the flow centerline, showing an increase from start to end for each flow rate condition. Our results, shown in Figure 4b, reveal similar trends along the centerline, indicating that the shear stress distribution on the magnesium samples aligns with the assumption of a uniform distribution. Optimizing the chamber design was essential to maintain laminar flow and prevent turbulence, which could lead to high shear stress peaks and localized corrosion [8,60]. Zhu et al. [28] performed CFD simulations indicated that shear stress and mass transfer coefficients increased with the flow rate, leading to a higher corrosion rate and more pronounced localized corrosion, particularly at the edges of the specimens. Our results, presented in Figure 5, demonstrate a consistent computational correlation, with higher shear stress values calculated for increased flow rates under comparable simulation conditions. Slight differences may be related to the use of different solutions with different rheological properties (SBF solution vs. demineralized water). Given our goal to investigate flow-induced corrosion under ocular conditions resulting from saccadic motions, demineralized water effectively simulates the ocular environment in elderly patients [51,56,59].

The primary aim of the computational models was to establish the correct relationship between the FISS field on the device’s external surfaces computed in Ferroni et al. [51] and the experimental inflow conditions needed to replicate the ocular environment. The SNOPT optimization algorithm was selected for its efficiency in handling large-scale problems. However, a comparison with the Nelder–Mead algorithm was also conducted. The results, as shown in Table 4, demonstrated the superior performance of the SNOPT approach.

The potential influence of fluid dynamics on pure Mg corrosion within a recreated ocular environment was thoroughly examined. The unique rheological ocular properties are a combination of reference fluid, temperature, and shear stress field, which together form an environment that well represents the state of pathological patients. This simplification on the characteristics of the fluid promoted a good correlation with the computational model, since the rheological properties of the liquefied vitreous are well approximated by demineralized water at 34 °C, fundamental for analyzing exclusively the flow-induced corrosion, avoiding further corrosion contributions.

Uniform corrosion on pure Mg samples increased with higher FISS values (Figure 9). This trend is primarily attributed to accelerated and controlled fluid movement, which enhances ion diffusion. Such increased diffusion affects the chemical equilibrium at the interface between the magnesium surface and the surrounding moving fluid, where corrosion phenomena are likely to occur [21,61]. Furthermore, research indicates that magnesium corrosion rates rise with increased mass transfer from the moving fluid [8,28,60].

Pre-existing localized superficial defects did affect the final corrosion pattern, but they were not directly caused by shear corrosion, in line with the evidences discussed by Zhu et al. [28]. In general, the study confirmed that the initial texture of the magnesium alloy significantly influences its corrosion resistance. Data from confocal laser scanning microscopy enabled the assessment of local height reductions and corresponding corrosion rates in an in vitro ocular environment. The CLSM analysis improved imaging results, allowing for a transition from qualitative observations to local and quantitative analysis of corroded surfaces. Specific post-processing algorithms were developed for this purpose. Comparisons were made between static and dynamic conditions, focusing on (i) height profile reductions, (ii) global corrosion rate analysis, and (iii) volume loss evaluations. Additionally, field emission scanning electron microscopy was used to acquire detailed images of the eroded surfaces.

The significant role of fluid dynamics in magnesium corrosion was well documented, highlighting the effects of fluid flow shear stress specific to the ocular system on specially manufactured Mg samples [27,28,49,60]. Analysis of surface morphologies after various FISS stimulations (Figure 6, top panel) revealed different corrosion patterns, including localized holes, MgO corrosion products, and flat, uniform corrosion. However, the presence of localized corrosion types, such as pitting corrosion, was strongly influenced by the pre-existing morphology of the samples (Figure 6, bottom panel). Despite this, the impact of local fluid flow conditions within the reference FISS range was minimal: in all cases, the fluid flow cleaned the surfaces of oxidized corrosion products, promoting uniform and flat erosion. This effect was more pronounced at higher FISS values, as previously noted by other researchers [8,49]. Higher FISS values than those studied here might lead to more severe localized corrosion, as increased FISS along the flow direction could accelerate the corrosion process [8].

The importance of uniform contribution to the corrosion mechanism was confirmed by CLSM data acquired at various timescales during biodegradation. CLSM data, when post-processed, showed a normal distribution of local height reduction across all tested FISS configurations (Figure 8). Higher FISS values led to greater height reductions and volume loss (VL), reaching a decrease of 2.85 μm after one day and an estimated VL of 1.7% of the initial volume after one week (FISS = 0.032 Pa).

The corrosion rate (CR) was analyzed in depth, with reliable CR values (3.435 μm/day) determined from CLSM data at 24–48 h under the most stressful in vivo conditions. For lower FISS values of 0.01 Pa and 0 Pa, the corresponding CR values were 2.7 and 1.9 μm/day, respectively. The CR values are in line with the results obtained by Wang et al. [8].

Moreover, it was observed that at higher shear stress values (FISS = 0.032 Pa), there is a notable washing-out effect of the corrosion products, which is not evident at lower shear stress conditions (Figure 8 and Figure 9). The removal of corrosion products at higher shear stresses suggests that the dynamic ocular environment, influenced by saccadic movements, plays a significant role in the corrosion mechanism of pure magnesium. These findings highlight the importance of considering shear stress variations when designing magnesium-based drug-delivery devices for ophthalmological applications, as the washing-out effect could influence the overall corrosion rate.

## 5. Conclusions

To design a new intravitreal device, we developed a combined experimental and computational approach to study the corrosion mechanisms of pure magnesium samples in an ophthalmological environment. We utilized a CFD model with an SNOPT optimization method to design experimental conditions that replicate the flow-induced shear stress (FISS) fields previously calculated for magnesium samples. Magnesium was selected due to its biocompatibility, biodegradability in biological fluids, and the ability to adjust its properties with alloys, making it a promising material for drug delivery in the vitreous cavity.

Corrosion tests were conducted, and the resulting morphological and profilometric data of the eroded magnesium surfaces were obtained using scanning electron microscopy (SEM) and confocal laser scanning microscopy (CLSM). These data were post-processed to assess corrosion rates. A thorough analysis was performed on the corrosion phenomena, including corrosion products, surface profile changes, corrosion rates over time, and volume loss. SEM images revealed instances of pitting corrosion, but a comparison of macro-morphologies before and after testing indicated that the growth of localized corrosion was not directly caused by the corrosion process itself. The uniform contribution to corrosion was confirmed by the normal distribution of local height reductions across all tested FISS configurations, as observed in CLSM data from various time points during the biodegradation process. The corrosion rates measured under three different FISS conditions (1.9, 2.7, and 3.435 μm/day) can be aligned with clinical requirements for drug release, providing a potential configuration for the device’s ocular applications.

Future studies may explore the effects of various alloying elements and surface treatments on the corrosion resistance of magnesium. Modifying the composition and surface properties might enhance the biocompatibility and corrosion performance of magnesium in ocular applications. Investigating the long-term corrosion behavior of magnesium samples in ophthalmological settings over extended periods is crucial. This could provide insights into the durability and performance of magnesium-based drug delivery devices for chronic ophthalmic treatments. Finally, studying the effects of different ocular conditions, such as variations in pH, saccades, and biochemical composition, on the corrosion behavior of magnesium could provide a more comprehensive understanding of its performance in real-world scenarios.

## Figures and Tables

**Table 1:** Values of Reynolds number Re when a parametric sweep of 7 imposed flow rate values Q is applied. The maximum Re is less than 1000 in the feasible region.

Q (mL/min)	Re
100	196
150	317
200	438
250	560
300	684
350	808
400	933

**Table 2:** Analysis of the performance of SNOPT method. The percentage difference between target and computed FISS values is always less than 0.1% in the feasible region.

τ_target_ [Pa]	Q_opt_ [mL/min]	τ_comp_ [Pa]	Time [min]	Δ [%]
0.01	154.26	0.010	87	0.050
0.03	240.94	0.030	102	0.000
0.05	317.28	0.050	140	0.002

**Table 3:** Computed flow rate Q values for known FISS targets.

τ_target_ [Pa]	Q_opt_ [mL/min]
0.008	143.5
0.01	154.3
0.023	212.8
0.032	248.3
0.04	279.4
0.046	294.6

**Table 4:** Comparison of performances between Nelder–Mead and SNOPT optimization approaches. SNOPT method shows results with higher accuracy in less simulation times.

Nelder–Mead				SNOPT		
τ_target_ [Pa]	Q_opt_ [mL/min]	τ_comp_ [Pa]	Time [min]	Q_opt_ [mL/min]	τ_comp_ [Pa]	Time [min]
0.01	52.025	0.001926	115	154.26	0.009995	87
0.03	240.9	0.029994	133	240.94	0.03	102
0.05	317.22	0.049987	220	317.28	0.050001	140

## Data Availability

The raw data supporting the conclusions of this article will be made available by the authors on request.
